# Supplementary material for: Nebulized and intravenous enzyme replacement therapy in mice with mucopolysaccharidosis type II
Source: PLoS One. 2025 Jun 26;20(6):e0310485. doi: 10.1371/journal.pone.0310485 (PMC12200698; doi:10.1371/journal.pone.0310485)
Supplement: Attachment — Submitted filename: PlosONE_MPSII_Response_Reviewers.docx [file pone.0310485.s004.docx]

# Dear Editor and Reviewers,

# Thank you for your assessment of our manuscript and constructive feedback. We have modified the text and figures to address all concerns and recommendations. The specific response to each comment is provided below.

# Sincerely,

# Leo

# Additional Editor Comments:

In your resubmission, specifically address the following:

- Relationship of the findings of the study, negative as they may be, with potential translation to humans.

**We have added a paragraph discussion the relationship of our findings to potential translation to humans**

- The percentage of drug which becomes available to the lungs, and contrast this with the effects seen in the liver.

**Unfortunately, we cannot establish the percentage with certainty. However, we have found in a previous study that the acute distribution is similar for lungs and liver. This may be due to exogenous IDS that remains circulating rather than taken up by the tissue. The sustained increase in activity occurred in the liver and not in the lungs. We have added text in the discussion (lines 358-365) to address this concern.**

- What, if any, human data exist in the literature or publicly available databases to provide supplemental support this manuscript.

**We have explained in the first paragraph of the discussion that human data from patients with MPS II are not available in the literature or public databases.**

- To what degree the proteomics analyses (pathway enrichments) explain the observations made.

**We have added text to the discussion for lines 342-347 outlining that the main difference from pathway enrichment that might explain the observations made in the liver are related to ‘peroxisomes’.**

[Note: HTML markup is below. Please do not edit.] Reviewers' comments:

Reviewer's Responses to Questions

# Comments to the Author

1. Is the manuscript technically sound, and do the data support the conclusions?

The manuscript must describe a technically sound piece of scientific research with data that supports the conclusions. Experiments must have been conducted rigorously, with appropriate controls, replication, and sample sizes. The conclusions must be drawn appropriately based on the data presented.

Reviewer **#1:** Yes

Reviewer #2: Yes

2. Has the statistical analysis been performed appropriately and rigorously?

Reviewer **#1:** Yes

Reviewer #2: Yes

3. Have the authors made all data underlying the findings in their manuscript fully available?

The PLOS Data policy requires authors to make all data underlying the findings described in their manuscript fully available without restriction, with rare exception (please refer to the Data Availability Statement in the manuscript PDF file). The data should be provided as part of the manuscript or its supporting information, or deposited to a public repository. For example, in addition to summary statistics, the data points behind means, medians and variance measures should be available. If there are restrictions on publicly sharing data-e.g. participant privacy or use of data from a third party­ those must be specified.

Reviewer #1: Yes

Reviewer #2: Yes

4. Is the manuscript presented in an intelligible fashion and written in standard English?

PLOS ONE does not copyedit accepted manuscripts, so the language in submitted articles must be clear, correct, and unambiguous. Any typographical or grammatical errors should be corrected at revision, so please note any specific errors here.

Reviewer #1: Yes

Reviewer #2: Yes

5. Review Comments to the Author

Please use the space provided to explain your answers to the questions above. You may also include additional comments for the author, including concerns about dual publication, research ethics, or publication ethics. (Please upload your review as an attachment if it exceeds 20,000 characters)

Reviewer #1: This is a well performed study investigating IDS-KO mice with idursulfase by combination of IV and nebulised form. It is difficult for me to directly comment on the methodology of inhalation & measurements of concentration in the mice as I have not performed such work before.

With regard to the key concepts and manuscript itself, I think it is an interesting and important study.

**Thank you.**

Do you think that this directly extrapolates to humans?

**Yes, we added a new paragraph to address this and other concerns and included statements to explain how the findings might extrapolate to humans (copied below and added in response to another comment below).**

**Lines 394-399:** *Importantly, nebulized ERT might be a feasible approach to bypass limitations of IDS crossing the blood brain barrier and reaching the central nervous system through axonal transport via the olfactory and trigeminal nerves (22-25). However, these topics were beyond the scope of our investigation. In general, our study suggests that nebulized IDS might be a safe, less invasive, and effective approach for ERT with potential to attenuate lung pathology in patients with MPS II disease.*

What% of this drug is absorbed into the lung using the nebuliser? It would be important to know if nebulising this drugs in humans would cause bronchoconstriction - can you tell us if there is any data in this area, and add a sentence on this in the conclusion.

**It is hard to define the percentage absorbed into the lung. Our study suggests there is no sustained absorption long-term (7 days post). However, the proteomics data suggest the enzyme is absorbed but since we do not see an increase in activity, we cannot establish the percentage. We have found in a previous study that acute distribution is similar for lungs and liver. This may be due to exogenous IDS that remain circulating rather than taken up by the tissue. The sustained increase in activity occurred in the liver and not in the lungs.**

**The concern with bronchoconstriction is difficult to address. We did not see signs of distress in our mice and the most important aspect is the mixing of IDS with an appropriate vehicle solution.**

**We have added text to address these concerns in the discussion (copied below, see also response to previous comment)**

**Lines 358-366:** *In a previous pilot study (20), we found that intravenous injection of IDS caused a similar increase in IDS activity in the lungs and liver of IDS KO mice when measurements were performed 2 hours post-injection. Therefore, it is reasonable to expect that the nebulized delivery increased lung IDS activity acutely and the increase exceeded that elicited by an intravenous injection such that lungs were exposed to a large percentage of the exogenous enzyme lungs initially. However, measurements performed within 2 hours post-injection might reflect circulating IDS rather than tissue uptake. Our current measurements performed seven days after ERT showed that IDS activity in the lungs did not increase with either IV treatment alone or the combination treatment with respect to the KO group.*

**Lines 380-394:** *Overall, the data suggests that exogenous IDS becomes available to the lungs acutely, but most of the long-term enzyme retention occurs in the liver. Regardless, ERT confers some improvement to the lung proteome within 4 weeks of treatment but changes in lung histology and function might require longer duration of treatment or more frequent dosing.*

*A potential concern with repeated dosing of exogenous IDS is the development of infusion-associated reactions, which have an allergenic nature and include bronchoconstriction (21). Although rare, adverse reactions are a concern and nebulization could exacerbate bronchoconstriction. There are no data in the literature to suggest that nebulized IDS per se would cause bronchoconstriction when applied in a solution with adequate osmolality. Mice in our study did not show signs of respiratory distress or behavioral discomfort when exposed to nebulized IDS. Future studies would have to test airway reactivity to IDS and pilot studies in patients might consider combining nebulized IDS with bronchodilators as precaution, which could even enhance benefits of nebulization when combined with lung volume recruitment strategies, e.g. positive end-expiratory pressure breathing.*

You have listed in the conclusion that you were unable to evaluate for any evidence of lung fibrosis. Is there any human data in this area? How do we know that improving activity in the liver and improving the molecular signature in the lungs will have a meaningful effect on patients?

**We have added information regarding evidence of lung fibrosis in older mice but have not found evidence in patients as it would require a biopsy and histology. However, we added a report of a patient that has clinical signs consistent with pulmonary fibrosis that were improved with short-term ERT. These are copied below.**

**Lines 368-371:** *However, a previous study has shown ERT via lentiviral vector increases IDS activity long-term in the lungs (21). We also did not detect changes in our histology markers of GAG or fibrosis in 5-month-old mice, but these were evident in 10-month-old mice (22).*

**Lines 377-380:** *This shows that there are disease and treatment effects on the tissue even though we could not detect elevated enzyme activity and a report in the literature is consistent with short-term pulmonary improvements in an infant receiving ERT (23).*

***Lines 385-388:*** *Overall, the data suggests that exogenous IDS becomes available to the lungs acutely, but most of the long-term enzyme retention occurs in the liver. Regardless, ERT confers some improvement to the lung proteome within 12 weeks of treatment but changes in lung histology and function might require longer duration of treatment or more frequent dosing.*

Reviewer #2: In the manuscript by Shamoun et al., the authors tested whether IDS administration using a nebulizer can increase IDS dosage in the lung in IDS-KO mice. However, there was no increase in IDS activity in the lung. The authors also perform proteomics analysis for GO and protein network using KEGG databases and identified some pathways

Although the authors' findings are not significant at all, this should not be a scope of this journal. The presented experiments are performed adequately, although the authors' analysis of the proteomics data remains shallow and limited. also, it was unclear whether the identified pathways by the proteomics analysis can explain the observations exactly. However, this is still acceptable because there may not be significance to perform proteomics analysis when there is no IDS increase in the lung with nebulization.

**Thank you for your comments. We have added information on how the identified pathway could explain the observations in the liver but being a discovery study, we cannot define with certainty. We decided to perform lung proteomics in the absence of a persistent increase in IDS activity based on our previous pilot observation that intravenous ERT increases IDS in lung samples and the possibility of an effect in the tissue that occurs with transient increases in IDS activity. We have added text in the discussion and throughout the manuscript to address these concerns (two key examples copied below).**

**Lines 358-366:** *In a previous pilot study (20), we found that intravenous injection of IDS caused a similar increase in IDS activity in the lungs and liver of IDS KO mice when measurements were performed 2 hours post-injection. Therefore, it is reasonable to expect that the nebulized delivery increased lung IDS activity acutely and the increase exceeded that elicited by an intravenous injection such that lungs were exposed to a large percentage of the exogenous enzyme lungs initially. However, measurements performed within 2 hours post-injection might reflect circulating IDS rather than tissue uptake. Our current measurements performed seven days after ERT showed that IDS activity in the lungs did not increase with either IV treatment alone or the combination treatment with respect to the KO group.*

**Lines 344-349:** *The ‘Peroxisomes’ pathway was downregulated in KO vs WT and KO-NEB, but not when compared to KO-IV. These data suggest that the absence of IDS in MPS-II decreases peroxisomes and ERT delivery via intravenous injection and nebulizer reached liver doses that restored abundance of proteins within peroxisomes and might have contributed to enhancement of hepatic lipid metabolism and diminish liver pathological features in MPS-II.*

The followings are minor comments.

Line 75 "multiple respiratory markers with ERT": explain "ERT" beforehand - i.e. describe "enzyme replacement therapy (ERT)" in Line 69

**Fixed**

Line 88 "Male wildtype C57BL/6": describe the substrain, C57BL/6N or C57BL/6J?

**Fixed**

Line 246: add "fibrosis judged by Masson's Trichrome staining"

**Added**

Line 247: explain what "percentage area stained for alcian blue" means

**We have added an explanation to the figure legend**

***Fig 2.*** *Quantification of markers of fibrosis analyzed by Masson’s Trichrome and glycosaminoglycans analyzed by Alcian Blue staining in lung and liver. Data shows area stained with Masson’s Trichrome or Alcian Blue expressed as a percentage of total tissue section*

All figures for the proteomics data: use larger fonts for readers

**We appreciate this concern and have tried larger fonts but could not fit the text with larger fonts and images in portrait mode. However, we changed the figures to landscape, which allowed us to increase font and image sizes.**
